# Mon4 as a novel monocyte subset with distinct profile and predictor of poor outcomes in individuals with myocardial infarction

**DOI:** 10.1007/s11239-025-03111-4

**Published:** 2025-05-26

**Authors:** Maxime Boidin, Gregory Y. H. Lip, Eduard Shantsila

**Affiliations:** 1 Liverpool Centre for Cardiovascular Science, University of Liverpool, Liverpool John Moores University and Liverpool Heart & Chest Hospital, Liverpool, UK; 2 School of Sport and Exercise Sciences, Liverpool John Moores University, Liverpool, UK; 3 Department of Sport and Exercise Sciences, Institute of Sport, Manchester Metropolitan University, Manchester, M1 7EL UK; 4 Danish Center for Health Services Research, Department of Clinical Medicine, Aalborg University, Aalborg, Denmark; 5 Primary Care, University of Liverpool, Liverpool, UK

**Keywords:** Major adverse cardiovascular events, Monocyte subsets, Myocardial infarction, Cardiac function

## Abstract

Recently, a new monocyte subset known as Mon4, characterized by distinct gene expressions, has been identified but remains poorly characterized. In this study, our objective was to comprehensively characterise Mon4 in healthy individuals and explore its correlation with major adverse cardiovascular events (MACE) in patients with ST-elevation myocardial infarction (STEMI). Our study enrolled 20 healthy individuals and 245 STEMI patients who underwent primary percutaneous coronary intervention (PCI). We analysed monocyte subsets using flow cytometry and collected bone marrow samples from 11 healthy individuals. Cardiac function assessments were performed in STEMI patients through echocardiography within 3 days post-PCI. Mon4 displayed significant differences compared to Mon1, Mon2, and Mon3 in various parameters among healthy individuals, underscoring its distinct profile. In STEMI patients, above-median Mon4 counts were associated with a increased risk of MACE (hazard ratio [HR] 3.11, 95% confidence interval [CI] 1.55–6.24, *p* = 0.01) and heart failure (HR 3.25, 95% CI 1.14–9.24, *p* = 0.03) after adjusting for other predictive factors. This study highlights the unique characteristics of Mon4 and its clinical significance. The distinctive gene signature of Mon4, coupled with its association with MACE and heart failure, suggests its potential utility as a biomarker for risk assessment in MI patients. Further investigations are warranted to explore the therapeutic potential of targeting Mon4 in reducing cardiovascular complications following MACE.

**Graphical Abstract:**

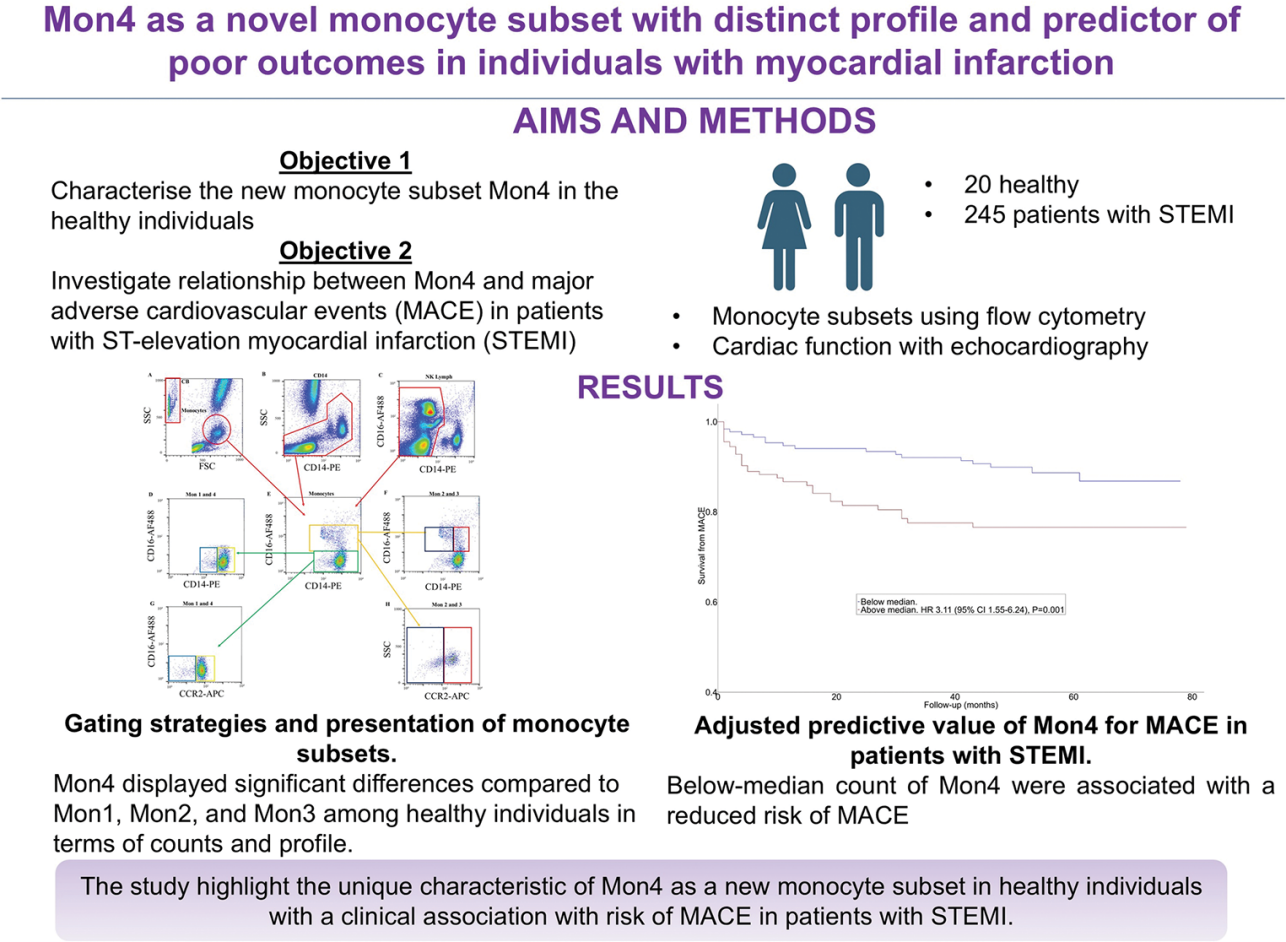

**Supplementary Information:**

The online version contains supplementary material available at 10.1007/s11239-025-03111-4.

## Introduction

Monocytes are a key component of the innate immune system and play a crucial role in immune surveillance, pathogen clearance, and immune regulation. Monocytes are not a homogenous population and include distinct subsets with diverse gene expression profiles and functional characteristics. Inflammation plays a crucial role in the development and progression of cardiovascular diseases, and emerging evidence suggests that specific subsets of monocytes, a key component of the innate immune system, are involved in this process (Libby, Ridker et al. [8], Wong, Tai et al. [23]). Understanding the unique properties of these monocyte subsets is essential for unraveling their contributions to immune responses and disease pathogenesis.

Monocytes are traditionally classified into three subsets based on surface marker expression: Mon1 (classical, CD14 + + CD16-), Mon2 (intermediate, CD14 + + CD16+), and Mon3 (nonclassical, CD14 + CD16++) (Wong, Tai et al. [23], Weber, Shantsila et al. [21]). Mon1, the most prevalent subset, is crucial for pathogen clearance and immune surveillance and are involved in patrolling the endothelium, phagocytosis, and antigen presentation. Mon2 is implicated in tissue repair, angiogenesis, cytokine production and in the progression of cardiovascular diseases, and are associated with a pro-inflammatory phenotype. Mon3 plays a role in immune regulation, tissue repair, inflammatory responses, extravasation into tissues, and potential roles in chronic inflammatory conditions (Wong, Tai et al. [23], Weber, Shantsila et al. [21]). Our group and other studies have shown that Mon1 (Berg, Ljungcrantz et al. [2]) and Mon2 (Tapp, Shantsila et al. [14], Shantsila, Ghattas et al. [12]) can independently predict adverse events in MI.

While classical, intermediate, and non-classical monocytes have been well-characterized, recent studies have shed light on the existence of a newly identified monocyte subset (Villani, Satija et al. [18], Vinci, Pedicino et al. [19]). This new subset has been revealed by Single-cell Ribonucleic acid (RNA)-sequencing and multicolour flow cytometry, and has been classified in various ways (e.g., intermediate monocyte subset, pre-classical monocyte subset, or natural killer dendritic cells) [6], Taieb, Chaput et al. [13], Welner, Pelayo et al. [22], Villani, Satija et al. [18], Merah-Mourah, Cohen et al. [9], Vinci, Pedicino et al. [19]). A study found that this new monocyte subset was present in individuals with non-ST elevation acute coronary syndrome (ACS) only (Villani, Satija et al. [18]). The new monocyte subset (abbreviated as Mon4 in this manuscript) exhibits distinct gene expression profiles with low expression of adhesion molecules CD49d, CD162, CD62L and of chemokine receptor CX3CR1 that could be indicative of functional changes or alterations in the activation state of monocytes (Merah-Mourah, Cohen et al. [9]). Other studies have highlighted the expression of genes associated with cytotoxic activity in immune cells (Villani, Satija et al. [18]). These characteristics suggest that Mon4 may have a distinct role in the pathogenesis of cardiovascular diseases.

Our study aimed to provide detailed description of Mon4 phenotype in healthy individuals and to investigate its relationship to clinical outcomes in patients with MI.

## Materials and methods

### Study design and participants’ recruitment

The analysis included 20 healthy individuals and 245 patients who were admitted with ST-elevation MI (STEMI) to National Health Service hospitals in Birmingham, United Kingdom: City Hospital (*n* = 84), Sandwell General Hospital (*n* = 32), Birmingham Heartlands Hospital (*n* = 107), and Queen Elizabeth Hospital Birmingham (*n* = 22) between November 2009 and November 2012, and met the study criteria. STEMI diagnosis followed the definition provided by the European Society of Cardiology, and all patients underwent primary percutaneous coronary intervention (PCI) (Van de Werf, Bax et al. [17]. Exclusion criteria for patients with STEMI were infectious diseases (e.g., sepsis) and inflammatory disorders (e.g., rheumatoid arthritis, psoriasis, and systemic lupus erythematosus) requiring treatment with steroids or other immunosuppressive agents, active cancer, severe renal failure, significant valvular heart disease, and previous myocardial infarction within the past six months. Patient data was excluded when flow cytometry quality standards were unmet (*n* = 13). Standard medical therapy post-PCI was administered to all study patients according to current European Society of Cardiology guidelines (Van de Werf, Bax et al. [17]. The study adhered to the Helsinki Declaration and obtained approval from the Coventry Research Ethics Committee (approval number 09/H1210/11). All participants provided written informed consent.

The healthy individuals reported to our laboratory between 11:00am and 12:00pm without fasting which were processed within 30 min of collection. Peripheral venous blood was collected after primary PCI within the first 24 h from admission (baseline). Blood samples of both groups were processed within 60 min for flow cytometric analysis of monocyte subsets. Plasma was obtained through centrifugation and stored at -70 °C for subsequent batched analyses. Flow cytometry, ELISA, and cardiac function measurements are detailed in supplemental (Supplemental material).

### Flow cytometry

**Fig. 1:**
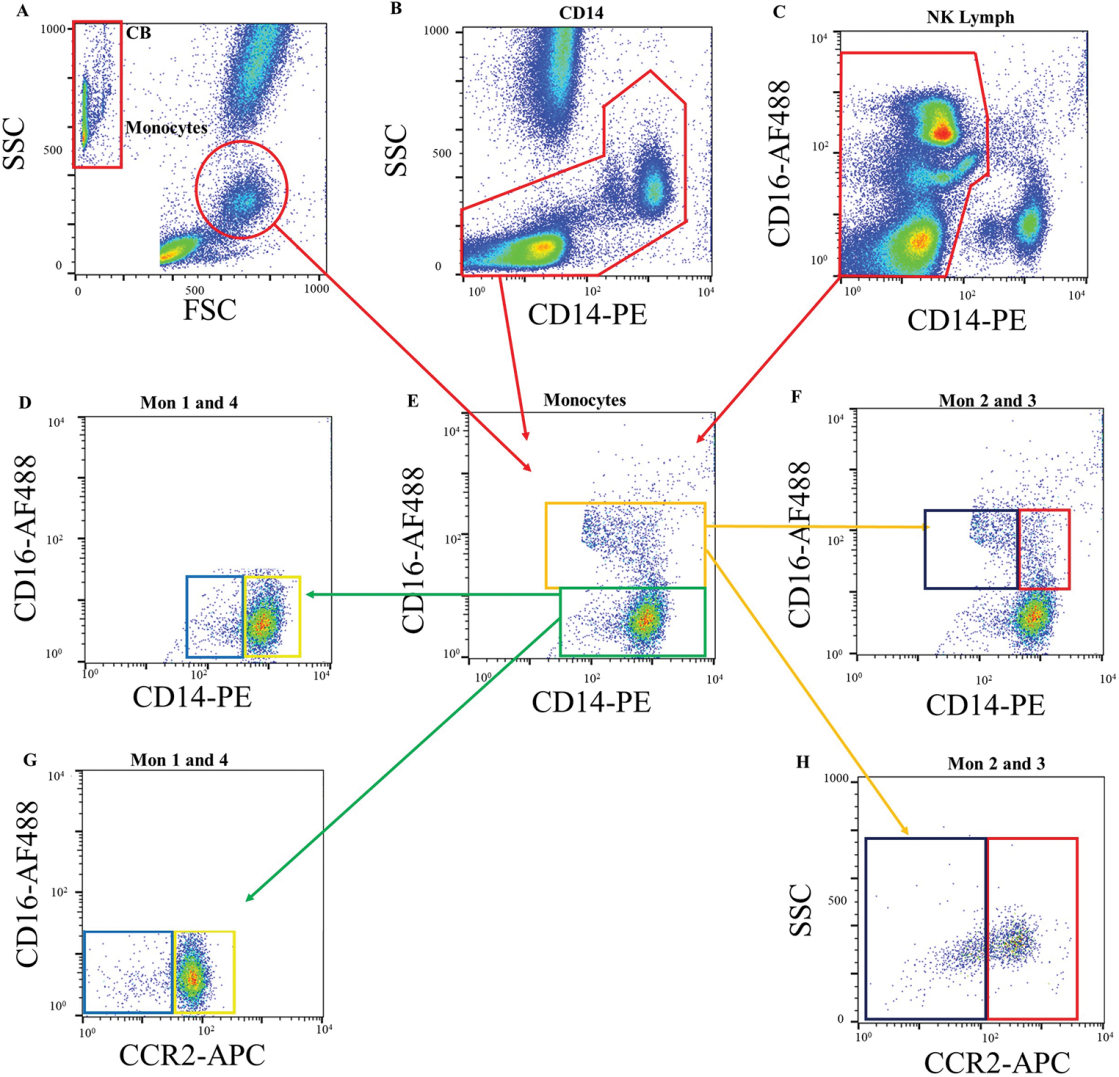
Gating strategies and presentation of monocyte subsets. (**a**) selection on monocyte region on forward scatter (FSC) vs. side scatter (SSC) plot, (**b**) separation of mononuclear cells from granulocytes, (**c**) selection of CD14–lymphocytes to separate CD16 + CD14– natural killer lymphocytes from CD16 + monocyte subsets, (**e**) separation of monocyte subset, that must correspond to regions Monos on (**a**) and Mon on (**b**) but must not include lymphocytes from (**c**), (**g**) separation of CD16 + monocyte subsets on the basis of their CCR2 expression, (**h**) CCR2 expression of Mon1, (**d**) and (**f**) location of Mon3 and Mon2, respectively, on CD14/CD16 plot. Mon 1: classical CD14 + + CD16-CCR2+, Mon 2: intermediate CD14 + + CD16 + CCR2 + and Mon 3: non-classical CD14 + CD16 + + CCR2– subsets

Monocyte subsets were quantified and characterised by flow cytometry. Flow cytometric analysis of monocyte subsets was conducted using a BD FACSCalibur flow cytometer (Becton Dickinson, Oxford, UK), following previously described methods (Shantsila, Wrigley et al. [11], Shantsila, Ghattas et al. [12]. The monocyte subsets were defined based on consensus guidance: Mon1 (‘classical’ CD14 + + CD16-), Mon2 (‘intermediate’ CD14 + + CD16 + CCR2+), and Mon3 (‘non-classical’ CD14 + CD16 + + CCR2-) (Weber, Shantsila et al. [21]) The new (Mon4) subset was defined CD14 + CD16- cells, which are seen as a small cluster of cells adjacent to Mon1 on CD14 vs. CD16 flow cytometry plot (Fig. 1). Monocyte platelet aggregates (MPAs) were defined as events positive to both monocyte markers (as above) and the platelet marker CD42a (glycoprotein IX). More details are available in Supplemental material.

### Outcome events

To answer our second research question, we investigated the first occurrence of major adverse cardiovascular events (MACE) in patients with STEMI only. MACE was defined as recurrent ACS (unstable angina, or non-STEMI, or STEMI with the presence of 2/3 criteria: that is, typical chest pain, electrocardiographic ischemic change, or elevated troponin T) (Thompson, Franklin et al. [16]), new clinical diagnosis of congestive heart failure (HF) based on symptoms and echocardiographic evidence of left ventricular dysfunction or death. The analysis excluded two cases of periprocedural death on the day of STEMI. Patients with STEMI were followed up using electronic hospital records from each recruitment site. Patients who were not reviewed in hospital were contacted to enquire about any events that were not recorded by their local hospital.

### Statistical analyses

Continuous data were reported as median and interquartile range (IQR) with Mann-Whitney unpaired test used to examine the differences between Mon4 and the other monocyte subsets (i.e., Mon1, Mon2, and Mon3) in the healthy group, and the baseline characteristics between individuals where MACE occurred versus those who did not experience MACE in the STEMI group. Univariate Cox proportional hazard ratios (HR) were calculated to evaluate the predictive value of Mon4 for survival outcomes in the study based on the median. Significant univariate predictors were included in multivariable models to determine their independent predictive value compared to the reference model. The reference model comprised age, sex, maximal troponin T levels, estimated glomerular filtration rate, and a history of hypertension and hypercholesterolemia, and smoking, and Mon2. Kaplan-Meier curves were constructed to assess survival based on relative medians. All statistical analyses were performed using R version 4.3.0 (PBC, Boston, MA, USA) with the following packages: survival [15] and survminer (Alboukadel, Marcin et al. [1]). P values < 0.05 were considered statistically significant.

## Results

### Blood Mon4 in healthy individuals

Baseline characteristics of healthy individuals have been published in our previous work (Shantsila, Wrigley et al. [11]. Mean ± SD age was 30 ± 6 years, body mass index was 23.2 ± 2.9 kg/m^2^, and 60% were women (Shantsila, Wrigley et al. [11]. Mon4 counts were 517 per µL, making it the scarcest monocyte subset. Mon4 phenotype showed significant differences compared to other subsets in their rates of aggregates with platelets (all *p* < 0.05). Mon4 was the smallest monocyte subset based on forward scatter (*p* < 0.001 vs. Mon1-Mon3), and showed least granularity compared to other subsets based on side scatter (*p* < 0.001 vs. Mon1-Mon3). Mon4 CCR2 expression was slightly lower than Mon1 (*p* = 0.02), similar to Mon2 (*p* = 0.08) and higher than on Mon3 (*p* < 0.001). Mon4 included a lower percentage with progenitor cells (*p* < 0.001 vs. Mon2-Mon3), and higher percentage compared to Mon1 (*p* < 0.001). Mon4 exhibited a higher expression of CD115, Tie2 and VEGF receptor 1 compared to Mon3 (all *p* < 0.001). Mon4 had lower expression of CCR2, CD64, and CD163 vs. Mon1 and Mon2 (all *p* < 0.001); of CD204, CXCR4, integrin β2, integrin α4, KDR, ferritin, ApoB, and TLR4 vs. Mon2 and Mon3 (all *p* < 0.001); of interleukin-6 receptor vs. Mon1 and Mon3 (all *p* < 0.05); and of CD34 vs. Mon1-Mon3 (all *p* < 0.001). More details are presented in Table 1.

**Table 1:** Clinical characteristics of the healthy individuals

	Mon1	Mon2	Mon3	Mon4	*P* values		
					Mon4 vs. Mon1	Mon4 vs. Mon2	Mon4 vs. Mon3
Count, cell/µL	650 (443–806)	90 (59–119)	60 (42–93)	52 (37–100)	**< 0.001**	**< 0.001**	**0.01**
Percentage of all monocytes, %	75 (62–81)	10 (7–12)	7 (5–11)	6 (4–13)	**< 0.001**	**< 0.001**	**0.001**
Aggregates with platelets, %	7.6 (5.6–11.7)	9.7 (7.3–14.3)	10.3 (8.2–13.6)	8.6 (6.4–12.3)	**< 0.05**	**0.003**	**< 0.001**
Forward scatter, channel	601 (578–630)	647 (616–680)	644 (617–686)	558 (529–594)	**< 0.001**	**< 0.001**	**< 0.001**
Side scatter, channel	346 (321–370)	354 (330–382)	356 (323–634)	291 (268–317)	**< 0.001**	**< 0.001**	**< 0.001**
CD14, MFI	1,727 (1,596-2,102)	1,856 (1,621-2,305)	177 (154–213)	559 (384–626)	**< 0.001**	**< 0.001**	**< 0.001**
CD16, MFI	5 (4–7)	44 (34–66)	114 (85–155)	4 (3–5)	**< 0.001**	**< 0.001**	**< 0.001**
CCR2+, %	99 (98–99)	97 (96–98)	60 (53–73)	94 (91–96)	**< 0.001**	**< 0.001**	**< 0.001**
CCR2, MFI	227 (180–269)	139 (110–193)	24 (21–33)	196 (127–237)	**0.02**	0.08	**< 0.001**
CD64, %	100 (100–100)	100 (99–100)	96 (89–98)	98 (97–99)	**< 0.001**	**< 0.001**	**0.002**
CD64, MFI	355 (324–407)	344 (277–368)	80 (69–93)	236 (199–292)	**< 0.001**	**< 0.001**	**< 0.001**
CD115+, %	41 (23–55)	84 (72–89)	80 (62–86)	14 (2–22)	**0.003**	**< 0.001**	**< 0.001**
CD115, MFI	15 (7–19)	40 (28–53)	34 (23–42)	9 (5–11)	**0.004**	**< 0.001**	**< 0.001**
CD204+, %	5 (3–16)	53 (41–75)	59 (52–70)	5 (2–15)	0.90	**< 0.001**	**< 0.001**
CD204, MFI	5 (4–9)	16 (13–59)	30 (23–48)	4 (4–7)	0.06	**< 0.001**	**< 0.001**
CD163+, %	98 (96–99)	96 (93–97)	72 (64–78)	83 (73–89)	**< 0.001**	**< 0.001**	**0.005**
CD163, MFI	201 (138–257)	255 (182–315)	36 (26–47)	54 (16–113)	**< 0.001**	**< 0.001**	0.05
Integrin α4+, %	37 (28–46)	75 (67–84)	81 (73–86)	27 (21–38)	**0.02**	**< 0.001**	**< 0.001**
Integrin α4, MFI	16 (13–19)	35 (28–41)	48 (42–55)	14 (12–18)	0.29	**< 0.001**	**< 0.001**
CXCR4+, %	46 (20–69)	78 (62–88)	66 (57–82)	46 (33–71)	0.56	**< 0.001**	**< 0.001**
CXCR4, MFI	18 (11–31)	43 (26–91)	53 (29–85)	20 (14–49)	0.17	**0.02**	**0.001**
Tie2+, %	3 (2–8)	41 (26–60)	53 (36–66)	5 (3–8)	0.18	**< 0.001**	**< 0.001**
Tie2, MFI	7 (6–10)	18 (12–24)	21 (13–32)	5 (4–5)	**< 0.001**	**< 0.001**	**< 0.001**
VEGF receptor 1+, %	6 (3–14)	49 (42–58)	49 (46–62)	3 (1–6)	**0.001**	**< 0.001**	**< 0.001**
VEGF receptor 1, MFI	7 (6–10)	18 (13–28)	21 (16–26)	5 (4–6)	**< 0.001**	**< 0.001**	**< 0.001**
KDR+, %	0 (0–2)	23 (23–23)	26 (15–44)	1 (0–3)	0.17	**< 0.001**	**< 0.001**
KDR+, MFI	3 (3–6)	5 (4–13)	8 (5–16)	3 (2–3)	**0.003**	**< 0.001**	**< 0.001**
CD34+, %	0 (0–0)	1 (1–1)	28 (21–36)	0 (0–1)	**< 0.001**	**< 0.001**	**< 0.001**
CD34+, MFI	3 (2–3)	3 (3–4)	4 (4–7)	2 (2–3)	0.21	**< 0.001**	**< 0.001**
Ferritin+, %	1 (0–2)	12 (5–15)	30 (21–36)	1 (0–2)	0.36	**< 0.001**	**< 0.001**
Ferritin, MFI	5 (4–6)	6 (5–7)	6 (5–9)	3 (3–4)	**< 0.001**	**< 0.001**	**< 0.001**
ApoB+, %	5 (2–14)	24 (16–40)	36 (30–43)	4 (2–13)	0.82	**< 0.001**	**< 0.001**
ApoB, MFI	6 (5–8)	10 (9–16)	14 (12–17)	5 (4–7)	0.09	**< 0.001**	**< 0.001**
TLR4+, %	5 (2–9)	40 (28–56)	44 (37–63)	3 (2–8)	0.41	**< 0.001**	**< 0.001**
TLR4, MFI	6 (5–8)	14 (10–20)	18 (12–35)	5 (4–6)	**< 0.001**	**< 0.001**	**< 0.001**
Interleukin-6 receptor, %	99 (95–100)	98 (92–99)	89 (80–93)	94 (82–97)	**< 0.001**	**0.003**	**0.03**
Interleukin-6 receptor, MFI	57 (48–65)	61 (53–72)	48 (39–57)	69 (49–82)	**< 0.05**	0.43	**0.001**
EPC, %	0.01 (0.00-0.04)	0.92 (0.92–0.92)	20.75 (13.48–26.20)	0.12 (0.03–0.39)	**< 0.001**	**< 0.001**	**< 0.001**
Mac-1, %	99.8 (99.5–99.9)	99 (97.9–99.6)	99.3 (98.5–99.7)	94.7 (88-97.3)	**< 0.001**	**< 0.001**	**< 0.001**
Mac-1, MFI	65 (51–253)	129 (93–289)	83 (72–124)	40 (31–118)	**0.004**	**< 0.001**	**< 0.001**

Data are expressed as median (IQR)

MFI: median fluorescence intensity; VEGF: vascular endothelial growth factor; KDR: killer lectin like receptor; ApoB: apolipoprotein B; TLR: toll-like receptors; EPC: endothelial progenitor cells; Mac-1: macrophage-1 antigen; IQR: interquartile ranges

In bold are parameters which were significantly different (*p* < 0.05)

### Mon4 in STEMI

Baseline characteristics of patients with STEMI have been published in our previous work (Shantsila, Ghattas et al. [12], Boidin, Lip et al. [4]). Among the 245 patients with STEMI (age 60 ± 12 years; 22% women), 82 (33%) developed a MACE during a median follow-up of 46 months. MACE included HF (*n* = 37, 45%), recurrent ACS (*n* = 34, 41%), and deaths (*n* = 19, 23%). Patients with MACE were older, had higher post-MI troponin T levels, lower eGFR, and a higher proportion of cardiovascular risk factors (*p* < 0.05 for all). Systolic and diastolic LV function, and global longitudinal strain were impaired in patients with STEMI who presented a MACE compared to those who did not (all *p* < 0.05). No difference was observed regarding flow cytometry parameters (Table 2).

**Table 2:** Characteristics of the study patients with STEMI on recruitment

	No MACE (*n* = 163)	MACE (*n* = 82)	Overall (*n* = 245)	*P* values
**Demographic and clinical characteristics**				
Age, years old	58 (51–66)	67 (58–73)	61 (52–68)	**< 0.001**
Female sex, n (%)	124 (76)	66 (80)	190 (78)	0.54
Body mass index, kg/m^2^	26 (25–29)	26 (24–27)	26 (25–28)	0.23
Current smokers, n (%)	96 (59)	41 (50)	137 (56)	0.22
Hypertension, n (%)	74 (45)	51 (62)	125 (51)	**0.02**
Type 2 diabetes, n (%)	31 (19)	26 (32)	57 (23)	0.07
Hypercholesterolemia, n (%)	45 (28)	38 (28)	83 (34)	**0.02**
Chronic obstructive pulmonary disease, n (%)	20 (12)	9 (11)	29 (12)	0.94
Cerebral vascular accident, n (%)	8 (5)	10 (12)	18 (7)	0.07
Myocardial infarction, n (%)	14 (9)	19 (23)	33 (13)	**0.003**
Percutaneous coronary intervention, n (%)	11 (7)	11 (13)	22 (9)	0.14
Coronary artery bypass surgery, n (%)	6 (4)	6 (7)	12 (5)	0.36
Inferior STEMI, n (%)	55 (34)	21 (26)	76 (31)	0.08
Left anterior descending STEMI, n (%)	36 (44)	44 (27)	80 (33)	**0.04**
**Biomarkers and flow cytometry parameters**				
Counts of Mon1, n	588 (456–742)	708 (563–977)	616 (488–841)	**< 0.001**
Counts of Mon2, n	106 (61–191)	156 (81–298)	118 (63–229)	**< 0.001**
Counts of Mon3, n	51 (33–71)	59 (43–82)	55 (37–72)	**0.03**
Counts of Mon4, n	30 (22–41)	38.5 (25-50.8)	32 (23–46)	**0.004**
Aggregates with platelets, %	8.0 (6.0–12.0)	8.5 (5.8–11.0)	8.0 (6.0–12.0)	0.68
Forward scatter, channel	689 (612–748)	721.5 (609–748)	699 (610–748)	0.63
Side scatter, channel	249 (226–274)	248.5 (227–275)	249 (227–275)	0.66
CD14, MFI	488 (387–591)	522 (384–635)	500 (386–599)	0.32
CD16, MFI	12 (10–14)	12 (10–13)	12 (10–14)	0.91
CCR2, MFI	129 (96–162)	128.5 (91–166)	129 (93–163)	0.88
Troponin T, ng/L	1958 (865–4550)	3930 (1692–9352)	2340 (1080–6020)	**0.001**
Creatine kinase, units/L	968 (435–1860)	985 (589–2370)	970 (455–2152)	0.37
eGFR, mL/min/1.73 m^2^	84 (65–90)	69 (56–84)	77 (63–90)	**< 0.001**
Total cholesterol, mmol/L	4 (4–5)	4 (4–5)	4 (4–5)	0.72
White cell counts, 10^9^/L	10 (8–12)	11 (8–13)	11 (8–13)	0.11
**Medications**				
RAAS inhibitors, n (%)	69 (42)	50 (61)	119 (49)	**0.008**
Diuretics, n (%)	22 (13)	19 (23)	41 (17)	0.18
Aspirin, n (%)	79 (48)	54 (66)	133 (54)	**0.01**
Beta-blockers, n (%)	46 (28)	30 (37)	76 (31)	0.23
Calcium channel blockers, n (%)	26 (16)	21 (26)	47 (19)	0.10
**Cardiac function**				
End-diastolic volume, mL	85.5 (72.8–98)	92 (82–107)	88 (74–102)	**0.01**
End-systolic volume, mL	38 (32–51)	45 (34-57.5)	41 (33.2–53)	**0.02**
Left ventricular ejection fraction, %	55 (47–59)	42 (36–52)	51 (43–57)	**< 0.001**
E/A	1 (0–1)	1 (0–1)	1 (0–1)	0.34
E/e’	9 (7–11)	10 (8–13)	9 (8–11)	**0.005**
Left atrial volume, mL	41 (33–51)	47 (34–58)	43.5 (33–53)	0.13
Fraction area change, %	32 (26–38)	35 (31–39)	32.5 (27–39)	0.08
TAPSE, mm	19.5 (17–21)	18.5 (17–22)	19 (17–21)	0.84
Global longitudinal strain, %	-16 (-17 - -13)	-11 (-14 - -9)	-14 (-16 - -11)	**< 0.001**
Global longitudinal strain rate, s^− 1^	154 (102–187)	135 (105–169)	143 (103–183)	0.43
Circumferential strain, %	-18 (-20- -13)	-12 (-17 - -10)	-15 (-20 - -11)	**0.003**
Circumferential strain rate, s^− 1^	147 (108-178.25)	153 (122-179.5)	148 (111–180)	0.58

Continuous variables are expressed as median (IQR); dichotomous variables are expressed as number and percentage

STEMI: ST elevation myocardial infarction; MACE: major adverse cardiovascular events; eGFR: estimated glomerular filtration rate; RAAS: renin-angiotensin-aldosterone system; IQR: Interquartile ranges

P values in bold when < 0.05 between No MACE and MACE groups

**Fig. 2:**
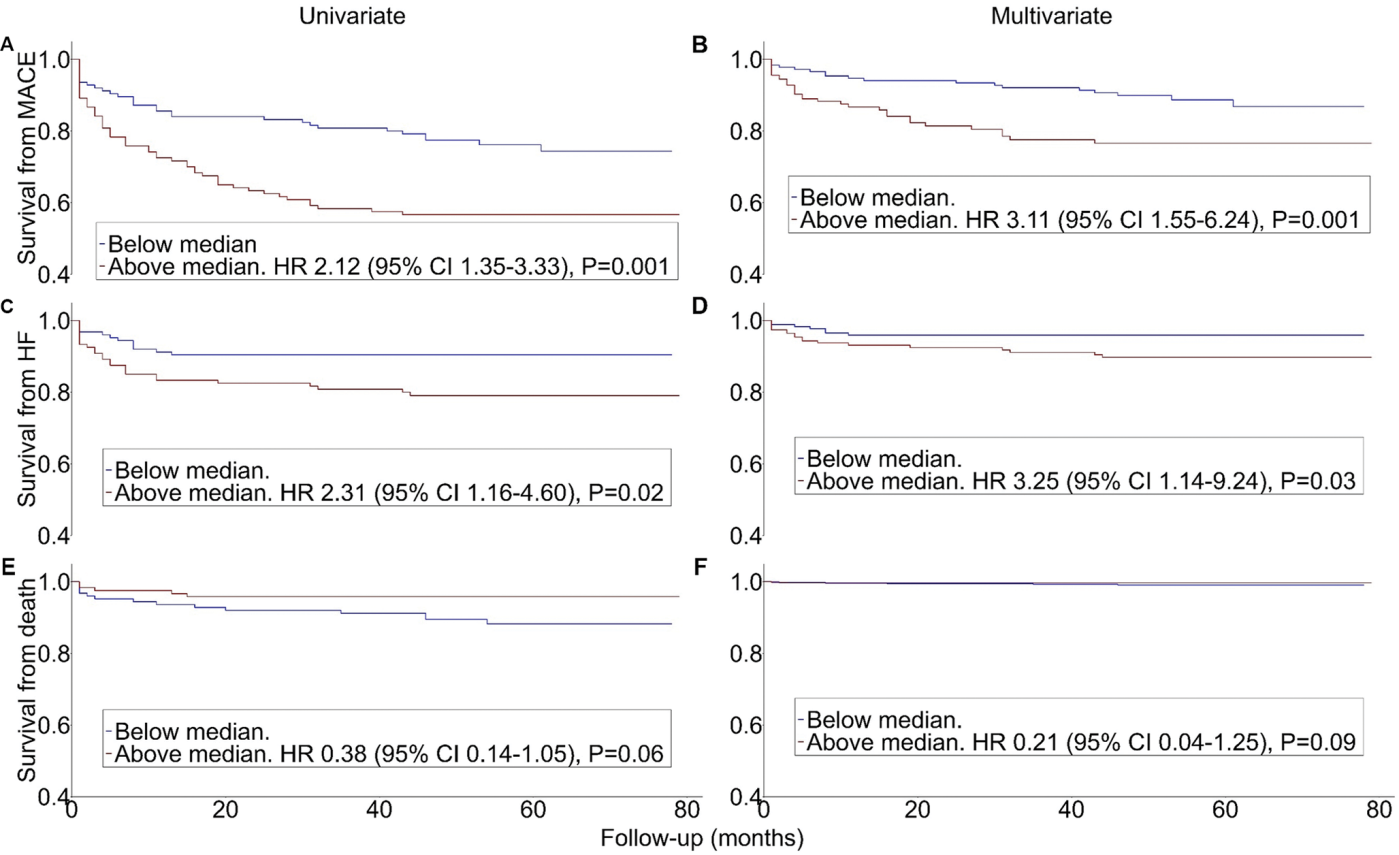
Predictive value of Mon4 for MACE, HF, and death. Univariate (left panels) and multivariate (right panels) **s**urvival analyses from MACE (panels **A** and **B**), HF (panels **C** and **D**), and death (panels **E** and **F**) in Mon4 according the baseline counts in blood counts of monocyte subsets. Multivariate analyses were adjusted for age, sex, maximal troponin T levels, estimated glomerular filtration rate, and a history of hypertension and hypercholesterolemia, smoking and Mon2. MACE: Major adverse cardiovascular events; HF: Heart failure; HR: Hazard ratio; CI: Confidence interval

On univariate analysis, above-median counts of Mon4 were associated with a 212% increased relative risk of MACE, and a 231% increased risk of HF compared to below-median counts of Mon4. After adjusting for the other predictors in multivariate analysis, above-median Mon4 levels remained significantly associated with a 311% increased risk of MACE and 325% increased risk of HF (Fig. 2).

## Discussion

In the context of MI, monocytes are crucial in orchestrating the inflammatory response and the different subsets may play a different role in the process. Although this new monocyte subset Mon4 has been already observed and classified in different ways, all agreed that it expresses a unique combination of genes that have the potential to affect their functions. Using flow cytometry, our study first time demonstrates distinct phenotype of Mon4 compared to Mon1, Mon2, and Mon3. The study further highlights the clinical significance of Mon4 as a potential biomarker for risk stratification in individuals with MI, suggesting its involvement in the pathophysiology of MACE and HF.

Monocytes play a crucial role in the immune response and inflammation by participating in various processes such as phagocytosis, antigen presentation, and cytokine production. The plasticity of monocytes to differentiate into different cell types highlights their versatility and their role in bridging the innate and adaptive immune responses, and their current gene expressions may vary over time, leading to different functions. This study demonstrates that Mon4 was already present in bone marrow, as it was the case in the other three subsets (Shantsila, Wrigley et al. [11]). Its presence in bone marrow means that Mon4 represents an independent subset rather than different stages of monocyte maturation.

This new Mon4 subset has been already observed in healthy individuals (Merah-Mourah, Cohen et al. [9]) and ACS patients without ST elevation (Vinci, Pedicino et al. [19]). Our study provides first data on its changes in STEMI and its impact for post-STEMI outcomes. This Mon4 observed in ACS patients with non-STEMI (Vinci, Pedicino et al. [19]) was linked the rupture of fibrous plaque cap and culprit plaque infiltration with macrophage. Macrophage infiltration represents is key for atherosclerotic plaque onset and progression and is closely related with systemic inflammation (Scalone, Niccoli et al. [10]). In the study with ACS patients with non-ST elevation, the levels of high-sensitivity C-reactive protein, which is a marker of inflammation where similar between those with intact fibrous caps and those ruptured fibrous caps, indicating a similar overall inflammatory burden in both patient groups (Vinci, Pedicino et al. [19]). However, the expansion of Mon4 was closely linked to macrophage infiltration and ruptured fibrous caps, while comparable frequencies were observed in intact fibrous caps and chronic coronary heart disease. These findings suggest that Mon4 plays a role in the pathogenesis of plaque rupture, independent to some extent from the systemic inflammation (Biasucci, Pedicino et al. [3]). We show Mon4 counts in acute STEMI are associated with clinical outcomes. This means that Mon4 may not only serve as a signature of plaque rupture or as a target for future therapeutic interventions but also as a powerful tool for clinical diagnosis, enhancing our ability to identify and manage patients at risk.

Activation of innate immunity plays a pivotal role in the pathogenesis of ACS by contributing to the development and progression of atherosclerotic plaques (Wang, Liu et al. [20]). Among the cells involved in innate immunity, monocytes stand out due to their remarkable plasticity and ability to adopt various functional phenotypes (Canè, Ugel et al. [5]). Studies have shown that approximately two-thirds of ACS patients, particularly those with plaque rupture identified through optical coherence tomography, exhibit signs of plaque macrophage infiltration and elevated systemic levels of inflammatory biomarkers (Scalone, Niccoli et al. [10]). Specifically, in ACS patients with ruptured fibrous caps, monocyte-derived macrophages demonstrate pro-thrombotic and inflammatory characteristics (Fracassi, Niccoli et al. [7]). The proportion of Mon4 is notably higher in ACS patients, particularly in those with ruptured fibrous cap and concurrent local macrophage infiltration (Vinci, Pedicino et al. [19]). It is worth emphasizing that macrophage infiltration represents a primary initiator of atherosclerotic plaque formation and progression. However, it’s important to note that not all ACS patients with plaque rupture exhibit local macrophage infiltration, and the presence of macrophages is closely associated with systemic inflammation (Scalone, Niccoli et al. [10]). Whilst systemic inflammation patterns are similar in non-ST elevation ACS patients with plaque erosion or rupture, the Mon4 is specifically linked to macrophage infiltration and the presence of ruptured fibrous caps. Additionally, a comparable frequency of Mon4 has been observed in non-ST elevation ACS patients with plaque erosion and those with chronic coronary syndrome (Vinci, Pedicino et al. [19]). This indicates that Mon4 has an important role in the pathogenesis of plaque rupture, independently from systemic inflammatory burden (Biasucci, Pedicino et al. [3]). Thus, in addition to the clinical marker of plaque rupture of Mon4, this monocyte can be also used as powerful predictor of cardiovascular complications in patients with STEMI.

This new identified Mon4 could serve as a potential biomarker for plaque rupture risk, offering a new diagnostic approach beyond traditional inflammatory indicators. In vitro experiments demonstrated that patients with non-ST elevation ACS with plaque rupture show distinct inflammatory responses, particularly in cytokine release, which may predict individual inflammatory reactions and potential clinical outcomes. Potential mechanisms need to be studied to elucidate the function of Mon4. This new monocyte appears to act as a critical inflammatory mediator, potentially serving as a sentinel cell that responds to early vascular injury and contributes to plaque destabilization (Villani, Satija et al. [18]). These findings suggest Mon4 as a promising target for future therapeutic interventions in cardiovascular disease, potentially providing insights into plaque progression and patient risk stratification.

The biological mechanism by which Mon4 might influence outcomes is not explored. Including more discussion on the potential functional roles of Mon4, supported by existing literature, would help.

### Strengths and limitations

Our study is the first to discriminate the specific characteristics of the new monocyte subset Mon4 using blood sample from the periphery. It is also the first to demonstrate the close relationship between Mon4 and MACE in large sample size (*n* = 245) of patients with STEMI. We used a robust, highly reproducible, and observer independent technique to analyse flow cytometry (Shantsila, Wrigley et al. [11]), we used different recruitment sites, which could lead to potential bias in terms of procedure. However, we used. The main limitation relates the observational nature of the study, that makes assessment of causality difficult. In patients with STEMI, the assessment of monocyte subsets was done using blood samples, which do not describe their behaviour in the myocardium. The study focused on monocyte counts and phenotype and assessment of their function was beyond the study scope.

## Conclusions

Our study sheds light on the significance of monocytes, particularly the newly described Mon4. Whilst Mon4 has been previously observed in different contexts, our study is the first to provide a detailed characterization of its unique phenotype in a healthy cohort, which differs from the other monocyte subsets. The study demonstrates the clinical significance of Mon4 as a potential biomarker for risk assessment in individuals with STEMI. We established a significant association between Mon4 coins and the risk of MACE and heart failure. Future studies are needed to better understand the mechanistic roles of Mon4 in people with MI and their potential as therapeutic targets.

## Electronic supplementary material

Below is the link to the electronic supplementary material.

Supplementary Material 1
