# Supplementary material for: Mon4 as a novel monocyte subset with distinct profile and predictor of poor outcomes in individuals with myocardial infarction
Source: J Thromb Thrombolysis. 2025 May 26;58(6):699–708. doi: 10.1007/s11239-025-03111-4 (PMC12350509; doi:10.1007/s11239-025-03111-4)
Supplement: Supplementary file 1 — Supplementary Material 1 [file 11239_2025_3111_MOESM1_ESM.docx]

**Supplemental material**

**Methods**

*Flow cytometry*

To perform the analysis, mouse anti-human monoclonal fluorochrome-conjugated antibodies were utilized, including anti-CD16-Alexa Fluor 488 (clone DJ130c; AbD Serotec, Oxford, UK), anti-CD14-PE (clone M/P9; BD), anti-CD42a-PerCP (clone Beb1; Becton Dickinson, Oxford, UK), and anti-CCR2-APC (clone 48607, R&D Systems, Abingdon, UK). These antibodies were mixed with 50 μL of freshly collected ethylenediaminetetraacetic acid (EDTA) anticoagulated whole blood in TruCount tubes (Becton Dickinson, Oxford, UK) containing a defined number of fluorescent count beads. After a 15-minute incubation period, the red blood cells were lysed using 450 μL of lysing solution (Becton Dickinson, Oxford, UK) for 15 minutes. Subsequently, the samples were diluted in 1.5 mL of Phosphate-Buffered Saline (PBS) and immediately subjected to flow cytometric analysis. The absolute count of each monocyte subset was calculated based on the acquired numbers of monocyte subsets and their proportion to the collected count beads, following the manufacturer's recommendations. Regular calibration of the flow cytometry is perform to maintain consistent fluorescence measurements. We followed robust quality control system, which included the use of fluorescent count beads (PE and FITC fluorochromes) in every blood sample processed. We monitored the position of count bead fluorescence on corresponding scales and redo samples that did not meet quality standards (e.g., observable deviation of fluorescence from the count beads). This technique is known for its robustness and high reproducibility, with a previous laboratory coefficient of variation of 1.9% for absolute monocyte count and <5% for surface markers (Shantsila, Wrigley et al. 2011).

*ELISA*

Matrix metalloprotease 9 (MMP9) and tumour necrosis factor-alpha (TNFα) levels were quantified only in patients with STEMI, using an enzyme-linked immunosorbent assay (ELISA) method. Frozen citrated plasma samples, stored at -70 °C, were used for batched analyses. Commercial ELISA kits known for their high quality were employed for this purpose: MMP9 (R&D Systems, Abingdon, UK) and TNFα (PeproTech, Rocky Hill, NJ, USA). The assays were performed following the manufacturer's instructions provided with the kits.

*Cardiac function*

Left ventricular ejection fraction (LVEF) was measured by biplane Simpson’s method using Philips iE33 echocardiography machine following current recommendations (Lang, Bierig et al. 2005). The scan was done within 3 days after presentation with acute STEMI, after PCI; Second harmonic imaging on a Philips iE33 machine with multifrequency phased- array transducer (1.5 MHz) was used to perform the studies. All measurements were made in triplicate and averaged according to the recommendations of the American Society of Echocardiography (Lang, Bierig et al. 2006). Studies were performed on the same model of the Philips machine across all sites.

**References**

Lang, R. M., M. Bierig, R. B. Devereux, F. A. Flachskampf, E. Foster, P. A. Pellikka, M. H. Picard, M. J. Roman, J. Seward, J. Shanewise, S. Solomon, K. T. Spencer, M. St John Sutton and W. Stewart (2006). "Recommendations for chamber quantification." Eur J Echocardiogr **7**(2): 79-108.

Lang, R. M., M. Bierig, R. B. Devereux, F. A. Flachskampf, E. Foster, P. A. Pellikka, M. H. Picard, M. J. Roman, J. Seward, J. S. Shanewise, S. D. Solomon, K. T. Spencer, M. S. Sutton and W. J. Stewart (2005). "Recommendations for chamber quantification: a report from the American Society of Echocardiography's Guidelines and Standards Committee and the Chamber Quantification Writing Group, developed in conjunction with the European Association of Echocardiography, a branch of the European Society of Cardiology." J Am Soc Echocardiogr **18**(12): 1440-1463.

Shantsila, E., B. Wrigley, L. Tapp, S. Apostolakis, S. Montoro-Garcia, M. T. Drayson and G. Y. Lip (2011). "Immunophenotypic characterization of human monocyte subsets: possible implications for cardiovascular disease pathophysiology." J Thromb Haemost **9**(5): 1056-1066.
